# Deletion of *Stk40* impairs definitive erythropoiesis in the mouse fetal liver

**DOI:** 10.1038/cddis.2017.148

**Published:** 2017-03-30

**Authors:** Lina Wang, Hongyao Yu, Hui Cheng, Ke He, Zhuoqing Fang, Laixiang Ge, Tao Cheng, Ying Jin

**Affiliations:** 1 Laboratory of Molecular Developmental Biology, Shanghai JiaoTong University School of Medicine, Shanghai 200025, China; 2 State Key Laboratory of Experimental Hematology, Institute of Hematology and Blood Disease Hospital, Chinese Academy of Medical Sciences and Peking Union Medical College, Tianjin 300020, China; 3 Center for Stem Cell Medicine, Chinese Academy of Medical Sciences, Tianjin, China; 4 Key Laboratory of Stem Cell Biology, CAS Center for Excellence in Molecular Cell Science, Institute of Health Science, Chinese Academy of Sciences, Shanghai 200032, China; 5 Department of Stem Cell & Regenerative Medicine, Peking Union Medical College, Tianjin, China

## Abstract

The serine threonine kinase Stk40 has been shown to involve in mouse embryonic stem cell differentiation, pulmonary maturation and adipocyte differentiation. Here we report that targeted deletion of *Stk40* leads to fetal liver hypoplasia and anemia in the mouse embryo. The reduction of erythrocytes in the fetal liver is accompanied by increased apoptosis and compromised erythroid maturation. *Stk40*^−/−^ fetal liver cells have significantly reduced colony-forming units (CFUs) capable of erythroid differentiation, including burst forming unit-erythroid, CFU-erythroid (CFU-E), and CFU-granulocyte, erythrocyte, megakaryocyte and macrophage, but not CFU-granulocyte/macrophages. Purified *Stk40*^−/−^ megakaryocyte–erythrocyte progenitors produce substantially fewer CFU-E colonies compared to control cells. Moreover, *Stk40*^−/−^ fetal liver erythroblasts fail to form normal erythroblastic islands in association with wild type or *Stk40*^−/−^ macrophages, indicating an intrinsic defect of *Stk40*^−/−^ erythroblasts. Furthermore, the hematopoietic stem and progenitor cell pool is reduced in *Stk40*^−/−^ fetal livers but still retains the multi-lineage reconstitution capacity. Finally, comparison of microarray data between wild type and *Stk40*^−/−^ E14.5 fetal liver cells reveals a potential role of aberrantly activated TNF-*α* signaling in *Stk40* depletion induced dyserythropoiesis with a concomitant increase in cleaved caspase-3 and decrease in Gata1 proteins. Altogether, the identification of Stk40 as a regulator for fetal erythroid maturation and survival provides new clues to the molecular regulation of erythropoiesis and related diseases.

Erythropoiesis, the biogenesis of red blood cells, is a complex morphogenetic process coordinated with lineage commitment and terminal differentiation. The final stages of erythropoiesis involve chromatin condensation, nuclear polarization and expulsion.^1, 2^ In mammals and some other vertebrates, the two types of erythropoiesis during embryonic development are defined as primitive and definitive erythropoiesis. The primitive erythropoiesis in the mouse initiates at E7.5 blood islands in the yolk sac. The yolk sac also produces erythroid/myeloid progenitors, which give birth to definitive erythroid cells.^3, 4^ Hematopoietic stem cells (HSCs) colonize the fetal liver soon after its origination at the aorta-gonad-mesonephros region at E10.5 and then migrate to the fetal liver around E11.5.^5, 6, 7^ The fetal liver since then is the dominant organ for the definitive erythropoiesis until birth. Burst forming unit-erythroid (BFU-E) and colony-forming unit-erythroid (CFU-E) subsequently expand exponentially during E14.5–E15.5 in numbers and generate definitive erythroid cells.^7^ Similar to erythropoiesis in adult bone marrow (BM), definitive erythroid precursors in the fetal liver also attach to macrophages to form erythroblastic islands (EBIs) and complete the terminal differentiation.^8^

For erythropoiesis, subsets of transcription factors have been defined as important regulators. Transcription factor GATA1 plays a central role in erythroid progenitor determination and differentiation.^9, 10^ Several cofactors, such as FOG1, TAL1 and LMO2, can interact with GATA1 and function in the regulation of erythroid-specific genes.^11, 12, 13, 14, 15^ EKLF/KLF1, another critical transcription factor, regulates erythroid maturation, hemoglobin switch and cytoskeleton homeostasis.^16, 17^ In addition, serine threonine kinases (STKs) were reported to play essential roles in erythropoiesis. For example, the STK activity is required for EKLF phosphorylation and function.^18^ Besides, the phosphatidylinositol 3-kinase/Akt mediates erythropoietin (Epo)-induced erythroid survival, proliferation and maturation.^19, 20^ REDK acts as a brake of erythropoiesis via phosphorylating myelin basic proteins as well as histone H2B and H3.^21^ Stk11 (LKB1) is well known for regulating cellular energy metabolism via activating AMPK and others, also being essential for quiescence and metabolic homeostasis of HSCs and erythropoiesis.^22^ However, the function of other STKs in erythropoiesis remains largely undefined.

Stk40 (serine/threonine kinase 40) was originally identified in our laboratory as a downstream target of pluripotency transcription factor Oct4 (encoded by *Pou5f1*) and shown to activate the ERK/MAPK pathway and induce extraembryonic endoderm differentiation in mouse embryonic stem cells.^23^ Our further studies have shown that targeted deletion of *Stk40* leads to mouse pulmonary defect and neonatal lethality,^24^ and that Stk40 represses adipogenesis by translational control of CCAAT/enhancer-binding proteins.^25^ The current study reveals that Stk40 can also function as a regulator of definitive erythropoiesis in the mouse fetal liver.

## Results

### *Stk40* knockout embryos suffer from anemia

**Figure 1:**
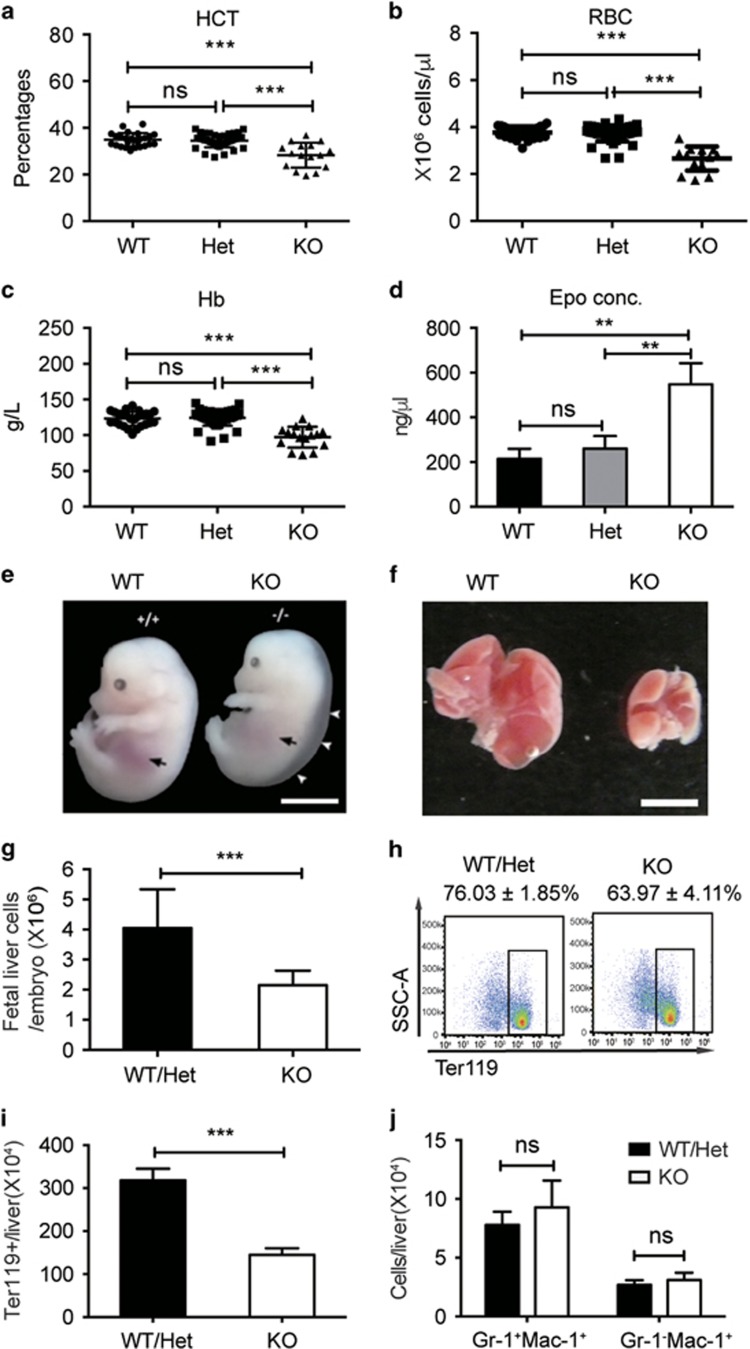
*Stk40* KO embryos have anemia. (**a**–**c**) Peripheral blood routine tests for E18.5 *Stk40* WT, Het and KO embryos. HCT, hematocrit; RBC, red blood cell; Hb, hemoglobin. WT, *n*=26; Het, *n*=48; KO, *n*=17. ***P≤0.001; ns, no significance. (**d**) Concentrations of erythropoietin (Epo) from *Stk40* WT, Het and KO embryos at E18.5. WT, *n*=6; Het, *n*=6; KO, *n*=6. **P≤0.01; ns, no significance. (**e**) Gross morphology of WT and *Stk40* KO embryos at E14.5. White arrowheads indicate subcutaneous edema. Black arrows indicate the position of the fetal liver. Scale bars, 5 mm. (**f**) Gross morphology of representative fetal livers from *Stk40* WT and KO embryos at E14.5. Scale bars, 2 mm. (**g**) Total number of fetal liver cells from *Stk40* WT/Het and KO embryos at E14.5. (**h**) The representative frequencies of Ter119^+^ cells from *Stk40* WT/Het and KO embryos at E14.5. (**i**) Absolute numbers of Ter119^+^ cells from *Stk40* WT/Het and KO embryos at E14.5. (**j**) Absolute numbers of monocytes (Gr-1^−^Mac-1^+^) and granulocytes (Gr-1^+^Mac-1^+^) from *Stk40* WT/Het and KO embryos at E14.5. For panels (**g**–**j**): WT, *n*=8; Het, *n*=30; KO, *n*=14. ***P≤0.001; ns, no significance

We previously reported that *Stk40*^−/−^ neonates died at birth with pale appearance and subcutaneous edema.^24^ We thus speculated that *Stk40*^−/−^ embryos might have anemia. To verify this, we examined the hematocrit of E18.5 embryos and found a moderate but significant decrease of hematocrit in the peripheral blood of *Stk40*^−/−^ embryos compared to heterozygous (Het) or wild-type (WT) specimens, while there was no significant difference in the hematocrit between *Stk40* Het and WT embryos (Figure 1a). Hereafter, WT and Het embryos were grouped together as WT/Het. The presence of anemia was further validated by reduced red blood cell counts and hemoglobin concentrations (Figures 1b and c). In contrast, counts of white blood cells and platelets were comparable between *Stk40*^−/−^ and WT/Het embryos (Supplementary Figures 1a and b). Significant decreases in the count of red blood cells were observed in *Stk40*^−/−^ embryos of E13.5–E18.5 (Supplementary Figure 1c). Moreover, the cell number to body weight ratios were also significantly reduced in *Stk40*^−/−^ embryos before birth (Supplementary Figure 1d). However, peripheral blood smears of *Stk40*^−/−^ embryos at E18.5 did not show obvious morphological abnormalities of erythrocytes (Supplementary Figure 1e). To test whether anemia in *Stk40* mutants was caused by an inadequate supply of Epo, an essential cytokine for erythrocyte production, we examined the concentration of Epo in the plasma of E18.5 embryos by ELISA. Interestingly, the Epo concentration in *Stk40*^−/−^ embryos displayed a twofold increase compared to that in WT/Het controls (Figure 1d). The finding implied that anemia observed in *Stk40*^−/−^ embryos was not caused by a low concentration of Epo. Instead, *Stk40*^−/−^ embryos might be under anemic stress to produce more Epo than normal controls. Together, these results reveal that loss of *Stk40* can lead to fetal anemia, suggesting a role of Stk40 in regulating erythropoiesis.

### Definitive erythropoiesis is impaired in *Stk40*^−/−^ fetal livers

The presence of anemia in *Stk40*^−/−^ embryos suggested that *Stk40* deletion could result in hematopoietic defects. The size of *Stk40*^−/−^ fetal livers was smaller than that of WT/Het fetal livers at E14.5 (Figures 1e and f), accompanied by significant decreases in the absolute and relative cellularity in the *Stk40*^−/−^ livers (Figure 1g, Supplementary Figures 1f and g). Flow cytometry analysis revealed that the frequency and absolute number of total erythroid cells (Ter119^+^) in the *Stk40*^−/−^ fetal liver decreased significantly (Figures 1h–i). Of note, the frequencies of fetal liver monocytes (Mac-1^+^Gr-1^−^) and granulocytes (Mac-1^+^Gr-1^+^) in *Stk40*^−/−^ embryos increased significantly compared to WT/Het controls, whereas the absolute number of these cells displayed no obvious difference between *Stk40*^−/−^ and WT/Het livers (Supplementary Figures 1h–i and Figure 1j). Furthermore, we did not detect significant differences in the frequencies of B220^+^ and CD3^+^ lymphocytes between *Stk40*^−/−^ and WT/Het embryos (Supplementary Figures 1j–k). As erythroid cells constitute the majority of the fetal hematopoietic compartment at this stage of development, our results suggest that the changes in the myeloid frequency are probably secondary to the erythroid reduction, supporting the notion that Stk40 is required for definitive erythropoiesis in the mouse fetal liver.

### Erythroid colony-forming progenitors decrease in *Stk40*^−/−^ fetal livers

**Figure 2:**
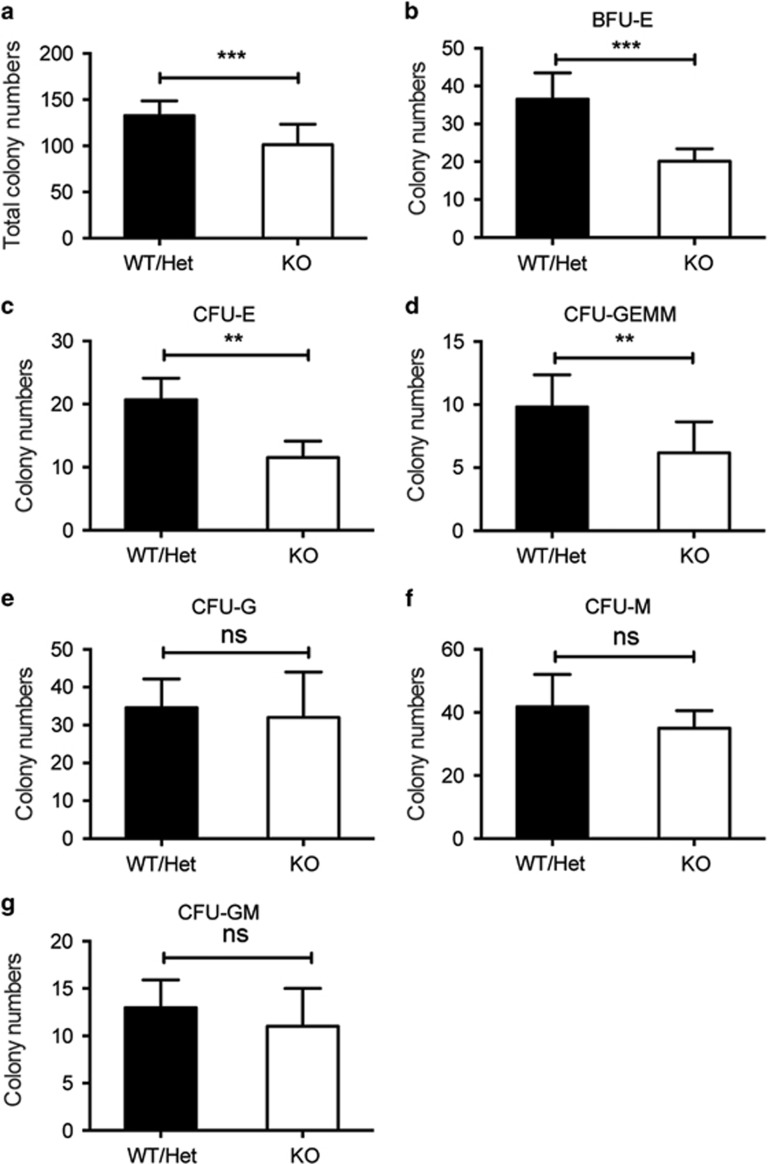
The number of erythroid colony-forming progenitors decreases in *Stk40*^−/−^ fetal livers. 2 × 10^4^ fetal liver cells from each E14.5 embryo were seeded in the media for colony-formation assays. Panels (**a**–**g**) show total colony numbers (**a**), BFU-E colony numbers (**b**), CFU-E colony numbers (**c**), CFU-GEMM numbers (**d**), CFU-G numbers (**e**), CFU-M numbers (**f**) and CFU-GM numbers (**g**) of *Stk40* WT/Het and KO fetal livers. WT, *n*=6; Het, *n*=12; KO, *n*=6. **P≤0.01, ***P≤0.001; ns, no significance

To determine whether *Stk40* depletion would affect hematopoietic progenitors, we analyzed colony-forming cells (CFCs) by culturing E14.5 fetal liver cells in the semisolid methylcellulose medium. Numbers of total CFCs and colonies containing erythroid cells (BFU-E, CFU-E and CFU-granulocyte, erythroid, macrophage and megakaryocyte (CFU-GEMM)) were all reduced substantially in *Stk40*^−/−^ livers compared to WT/Het ones (Figures 2a–d and Supplementary Figures 2a–c). In contrast, numbers of myeloid CFCs (CFU-granulocyte (CFU-G), CFU-macrophages (CFU-M) and CFU-granulocyte/macrophages (CFU-GM)) were not significantly affected by *Stk40* deletion (Figures 2e–g and Supplementary Figures 2d–f), indicating that the decrease in the mix-lineage colony number could be attributed to the impaired production of erythroid cells. These results suggest that Stk40 is required for the generation and/or maintenance of erythroid progenitor cells from HSCs.

### *Stk40* deletion leads to a reduced fetal liver hematopoietic stem and progenitor cell pool

**Figure 3:**
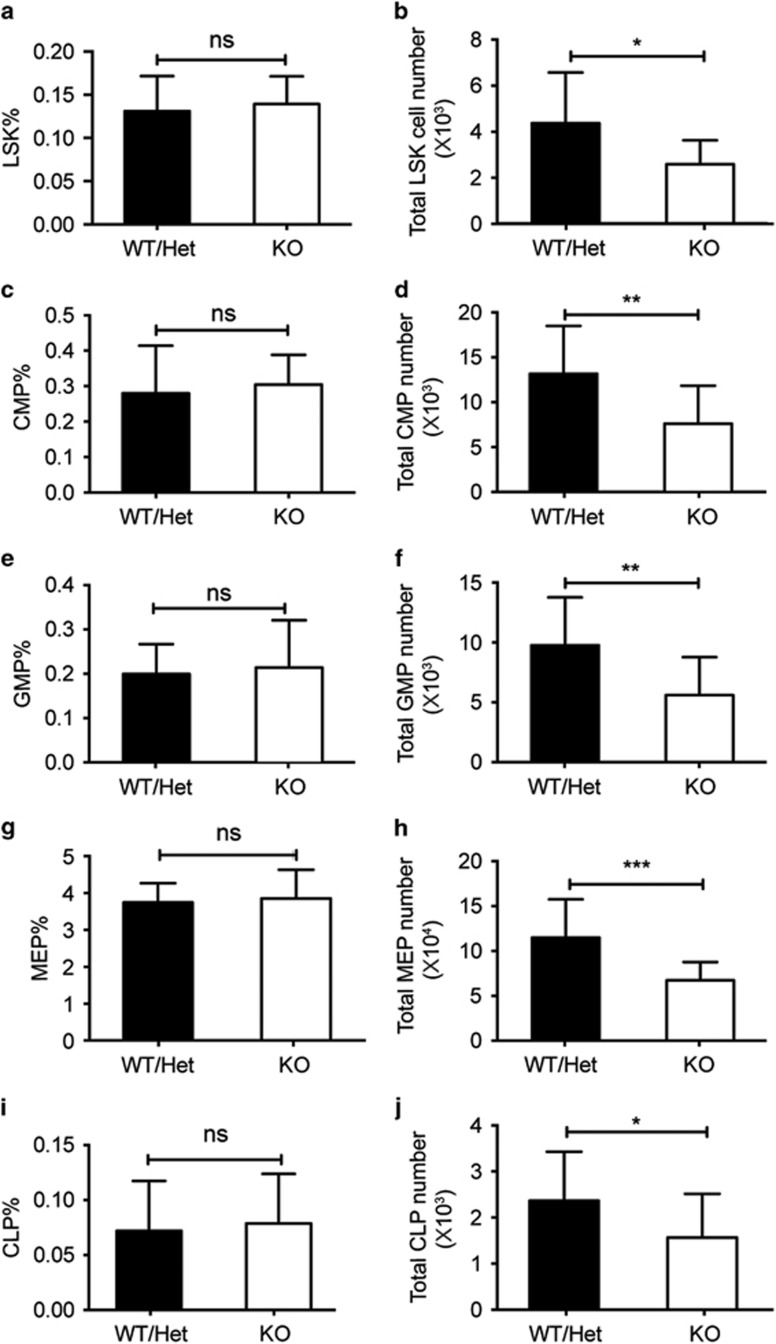
Numbers of HSPCs are reduced in *Stk40*^−/−^ fetal livers. (**a** and **b**) Frequencies and absolute numbers of LSK cells of each E14.5 *Stk40* WT/Het and KO fetal liver. (**c** and **d**) Frequencies and absolute numbers of common myeloid progenitor of each E14.5 *Stk40* WT/Het and KO fetal liver. (**e** and **f**) Frequencies and absolute numbers of granulocyte–monocyte progenitor of each E14.5 *Stk40* WT/Het and KO fetal liver. (**g** and **h**) Frequencies and absolute numbers of MEP of each E14.5 *Stk40* WT/Het and KO fetal liver. (**i** and **j**) Frequencies and absolute numbers of common lymphoid progenitor of each E14.5 *Stk40* WT/Het and KO fetal liver. For panels (**a**–**j**), WT/Het, *n*=27; KO, *n*=18. *P≤0.05, **P≤0.01, ***P≤0.001; ns, no significance

To determine how *Stk40* deletion impaired erythropoiesis, we evaluated the effect of *Stk40* depletion on frequencies of hematopoietic stem and progenitor cells (HSPCs) in the fetal liver. Results from flow cytometry analysis indicated that the frequencies of various HSPC populations were not affected by *Stk40* deletion, including HSC-containing Lin^−^Sca1^+^c-Kit^+^ (LSK) cells, common myeloid progenitors (Lin^−^Sca1^−^c-Kit^+^CD34^+^CD16/32^lo^), granulocyte–monocyte progenitors (Lin^−^Sca1^−^c-Kit^+^CD34^+^CD16/32^hi^), megakaryocyte–erythrocyte progenitors (MEPs, Lin^−^Sca1^−^c-Kit^+^CD34^−^CD16/32^−/lo^) and common lymphoid progenitors (Lin^−^IL-7R*α*^+^Sca1^+^c-Kit^+^) (Figures 3a, c, e, g, i and Supplementary Figure 3). The finding was consistent with the result of colony-forming assays. However, absolute numbers of various HSPCs were significantly fewer in *Stk40*^−/−^ fetal livers than those in controls (Figures 3b, d, f, h and j) probably due to the reduced total cellularity of *Stk40*^−/−^ fetal livers, indicating the defective hematopoiesis caused by *Stk40* deletion.

**Figure 4:**
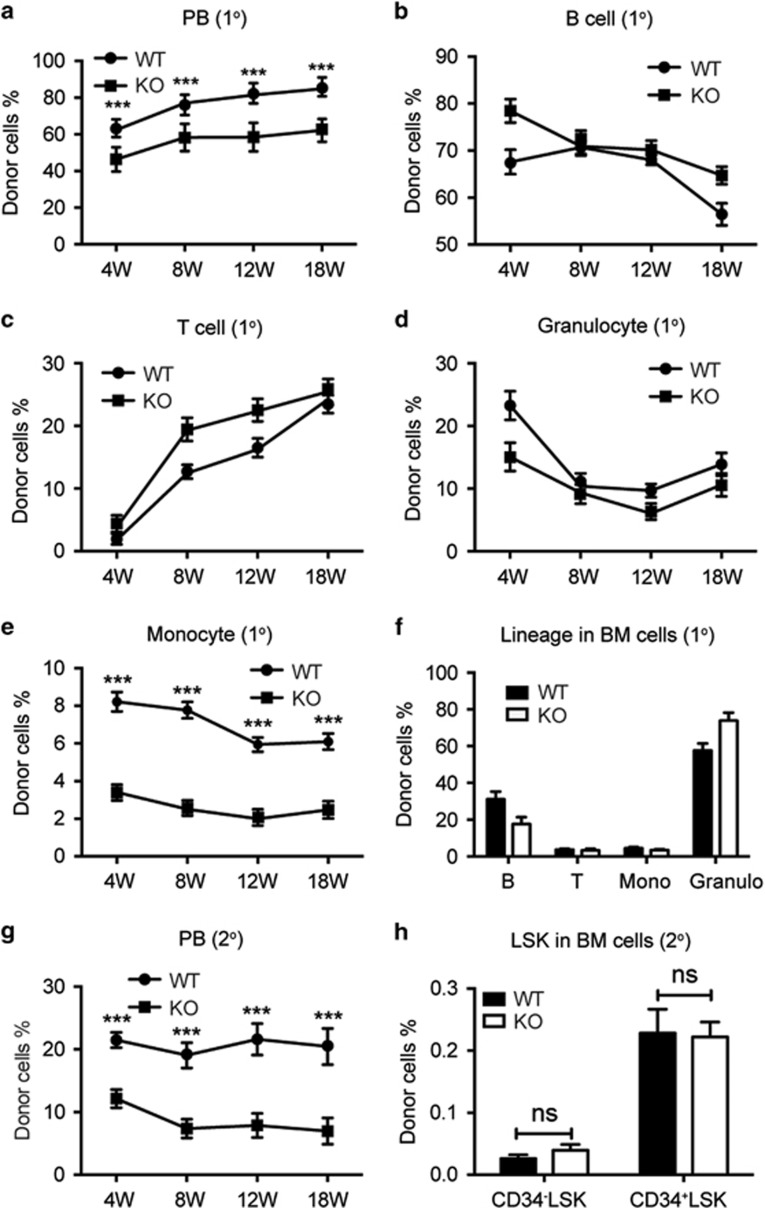
*Stk40*^−/−^ fetal liver cells retain the long-term multi-lineage reconstitution capacity. (**a**) Donor-derived cell chimerism from WT and *Stk40* KO fetal livers at different weeks after the primary competitive reconstitution. 1°, primary transplantation; PB, peripheral blood. (**b**–**e**) Frequencies of B lymphoid (B220^+^, **b**), T lymphoid (CD3^+^, **c**), granulocyte (Gr-1^+^Mac-1^+^, **d**) and monocyte (Gr-1^−^Mac-1^+^, **e**) in donor (CD45.2^+^) derived cells in the peripheral blood from the primary competitive reconstitution assay. (**f**) Frequencies of donor derived lineages in BM cells at 18 weeks after the primary competitive reconstitution assay. (**g**) Donor-derived cell chimerism of PB at different weeks after the secondary transplantation (2°). (**h**) Frequencies of donor-derived LSK cells in BM at 18 weeks after the secondary transplantation. For the primary transplantation, WT, *n*=10; KO, *n*=14. For the secondary transplantation, *n*=12 for each group. ***P≤0.001; ns, no significance

To determine the functionality of *Stk40*^−/−^ HSCs, we performed competitive reconstitution assays with E14.5 fetal liver cells mixed with BM competitor cells. The donor cell chimerism of *Stk40*^−/−^ fetal livers was lower than that of WT cells in the primary transplantation (Figure 4a), suggesting a defect in the hematopoietic repopulation of *Stk40*^−/−^ HSCs. The lineage compositions of B, T lymphocytes and granulocytes derived from the donor cells were normal (Figures 4b–d). However, the frequency of monocytes was significantly lower than controls (Figure 4e). Hence, *Stk40*^−/−^ fetal liver cells retained the capacity of multi-lineage reconstitution. In addition, the chimerism of *Stk40*^−/−^ donor-derived cells in the BM was reduced compared to WT donor cells (Supplementary Figure 4a), while the multi-lineage composition in the BM was not obviously altered (Figure 4f).

In the secondary transplantation, the donor-derived cell chimerism of *Stk40*^−/−^ transplants remained significantly lower than WT controls, although the lineage composition of donor-derived cells in the peripheral blood was comparable, except for monocytes (Figure 4g and Supplementary Figures 4b–e). Interestingly, the ratio of average donor chimerism of *Stk40*^−/−^ HSCs compared to WT cells was further lessened (Supplementary Figure 4f), which is coincident with the reduced donor chimerism in the BM (Supplementary Figures 4a and g). Nevertheless, the frequencies of CD34^−^LSK and CD34^+^LSK cells, which represent long-term HSCs and short-term HSCs/multipotent progenitors, respectively, were not significantly altered (Figure 4h). Moreover, the frequencies of progenitors, including common myeloid progenitor, granulocyte–monocyte progenitor and MEP, were comparable between *Stk40* WT and KO donor-derived cells (Supplementary Figure 4h). Therefore, the less contribution of *Stk40*^−/−^ transplants was probably due to the reduced size of functional HSC pool in the *Stk40*^−/−^ fetal liver. Unfortunately, we could not evaluate the donor-derived erythropoiesis due to the lack of markers to distinguish the origin of erythroid cells. These results indicate that *Stk40* deletion leads to a reduction of functional HSC populations in the mouse fetal liver but does not abolish the capacity of fetal liver HSCs for long-term multi-lineage reconstitution.

### *Stk40* deletion impairs erythroid differentiation, maturation and survival in the fetal liver

**Figure 5:**
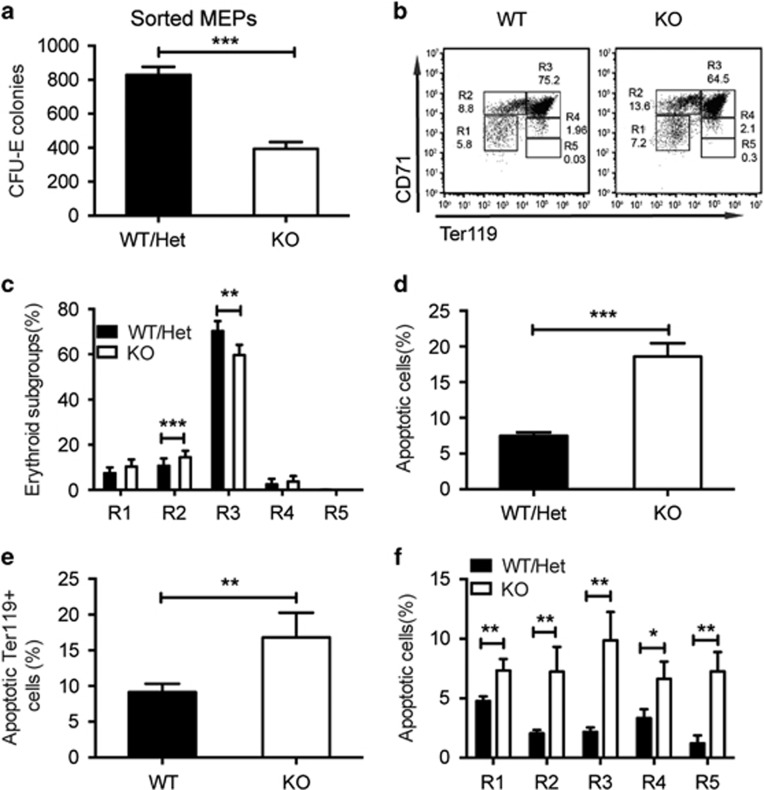
Impaired erythroid maturation and increased apoptosis in *Stk40*^−/−^ fetal livers. (**a**) CFU-E colony numbers of purified MEPs from *Stk40* WT/Het and KO embryos at E14.5. 2 × 10^3^ freshly sorted MEPs of E14.5 fetal liver cells were seeded in the media for colony-formation assays. WT, *n*=8; KO, *n*=8. ***P≤0.001. (**b**) Representative flow cytometry profiles of R1–R5 from E14.5 *Stk40* WT/Het and KO mice with antibodies against CD71 and Ter119, respectively. (**c**) Frequencies of cells in each R population (from R1 to R5), normalized to the number of total viable fetal liver cells from *Stk40* WT/Het and KO mice. WT/Het, *n*=19; KO, *n*=13. **P≤0.01, ***P≤0.001. (**d**) Frequencies of apoptotic cells based on Annexin V^+^PI^−^ cells of total fetal liver cells of E14.5 *Stk40* WT/Het and KO embryos. WT/Het, *n*=24; KO, *n*=14. ***P≤0.001. (**e**) Frequencies of apoptotic cells within the Ter119^+^ population of fetal liver cells of E14.5 *Stk40* WT/Het and KO embryos. WT/Het, *n*=14; KO, *n*=6. **P≤0.01. (**f**) Frequencies of apoptotic cells within each R population ranging from R1 to R5 of fetal liver cells of E14.5 *Stk40* WT/Het and KO embryos. WT/Het, *n*=14; KO*, n*=6. *P≤0.05, **P≤0.01

We next examined whether *Stk40* deletion would affect functions of MEPs in addition to reducing the population of MEPs in fetal livers. Equal numbers of purified MEPs of E14.5 fetal livers from both *Stk40* WT/Het and KO embryos were used for CFC assays. *Stk40*^−/−^ MEPs formed considerably fewer CFU-E colonies (Figure 5a). However, there was no obvious difference in the percentage of apoptotic MEPs between *Stk40*^−/−^ and WT/Het embryos (Supplementary Figures 5a and b). This finding suggested that Stk40 is required for MEPs to function properly.

To investigate whether the absence of *Stk40* would also affect erythroid terminal differentiation, we analyzed erythroid maturation of E14.5 fetal liver cells by flow cytometry analysis using CD71 and Ter119 antibodies. In *Stk40*^−/−^ fetal livers, pro-erythroblasts and early basophilic erythroblasts (R2, CD71^+^Ter119^−^) significantly increased, while the early and late basophilic erythroblasts (R3, CD71^+^Ter119^+^) decreased, indicating a partial blockage of erythroid maturation (Figures 5b and c). Furthermore, impaired erythropoiesis is often accompanied by increased cell death. Indeed, in line with the reduced cellularity and erythropoiesis, we detected an over twofold increase in apoptotic cells in *Stk40*^−/−^ fetal livers determined by Annexin V staining (Figure 5d). Importantly, the percentages of apoptotic Ter119^+^ cells and all subgroups (R1-R5) of erythroid cells increased significantly in *Stk40*^−/−^ fetal livers compared to those in WT/Het controls (Figures 5e and f). These data indicate that Stk40 is important for erythroid cell survival and lineage differentiation from MEPs.

### Stk40 is required for normal EBI formation

**Figure 6:**
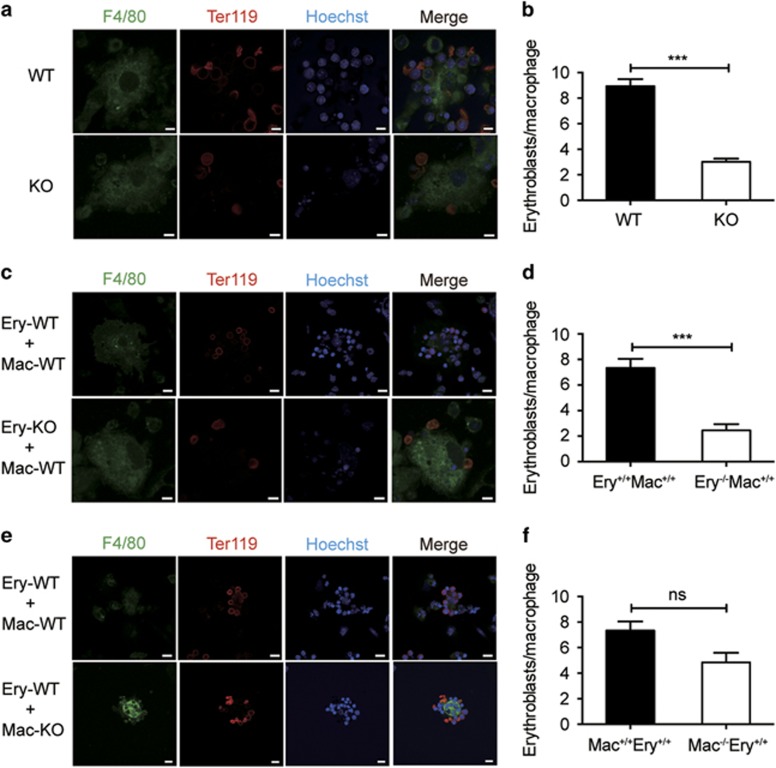
Stk40 is crucial for the EBIs formation. (**a**) Representative images of native EBIs isolated from fetal livers of E14.5 WT and *Stk40* KO embryos. Islands were stained with F4/80-FITC (green) for macrophages, Ter119-APC (red) for erythroblasts, and Hoechst 33342 for the nuclei. Scale bar, 5 *μ*m. (**b**) Numbers of erythroblasts bound per macrophage of E14.5 WT and *Stk40* KO embryos. Sixty islands from nine embryos were counted for each genotype. ***P≤0.001. (**c**) Representative images of reconstituted islands from WT and *Stk40* KO erythroblasts (Ery) incubated with WT macrophages (Mac) of E14.5 WT and *Stk40* KO embryos. (**d**) Numbers of erythroblasts bound per macrophage. For each cell combination, total 40–60 islands from 10 embryos were counted. ***P≤0.001. (**e**) Representative images of reconstituted EBIs from WT erythroblasts (Ery) incubated with WT and *Stk40* KO macrophages (Mac), respectively. (**f**) Numbers of erythroblasts bound per macrophage. For each cell combination, total 40–60 islands from 10 embryos were counted. Ns, no significance. For panels (**c** and **e**), scale bar, 10 *μ*m

The erythroblasts in the fetal liver differentiate in association with centrally positioned macrophages and form EBI, which play an important role in erythroblast maturation.^26, 27, 28^ To investigate how *Stk40* deficiency led to defects in erythroid cells, we conducted EBI formation assays to compare native EBIs isolated from WT E14.5 fetal livers to those from *Stk40*^−/−^ embryos. We found that the number of erythroblasts associated with each macrophage was significantly decreased in the native islands of *Stk40*^−/−^ fetal livers (Figures 6a and b), suggesting an essential role of Stk40 in the EBI formation. In addition, we also carried out reconstituted EBI formation assays to determine the contribution of defects in macrophages and/or erythroblasts to the aberrant EBI formation. *Stk40*^−/−^ erythroblasts were substantially less effective than WT erythroblasts in forming association with macrophages (Figures 6c and d). On the other hand, compared to WT macrophages, *Stk40*^−/−^ macrophages bound slightly fewer WT erythroblasts without statistical significance (Figures 6e and f). These observations indicate that impaired EBI formation in *Stk40*^−/−^ fetal livers was mainly due to defects in erythroblasts, although alterations in macrophages cannot be entirely excluded. Moreover, typical rosette organization of EBIs was easily observed in WT livers, but hardly found in *Stk40*^−/−^ livers by transmission electron microscopic examination (Supplementary Figure 6). Therefore, Stk40 is critical for erythroblast/macrophage association during erythroid maturation.

**Figure 7:**
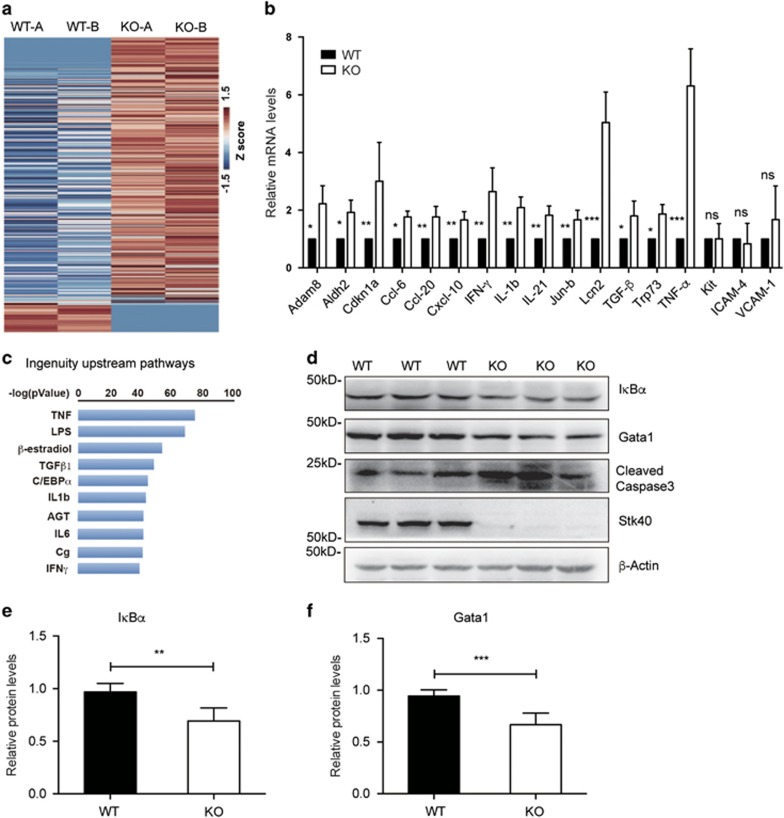
Stk40 regulates TNF-*α* signaling and genes involved in erythropoiesis. (**a**) The heat map shows DEGs of E14.5 WT and *Stk40* KO fetal liver cells based on RNA microarray data. (**b**) Expression levels of some selected DEGs in the E14.5 fetal liver cells were verified by RT-qPCR. WT, *n*=8; KO, *n*=8. (**c**) Upstream pathways of upregulated genes in E14.5 *Stk40* KO fetal liver cells compared to WT controls were identified by the ingenuity pathway analysis. (**d**) Representative western blot analysis shows protein levels of I*κ*B*α*, Gata1, cleaved caspase-3, Stk40 and *β*-actin in E14.5 WT and *Stk40* KO fetal liver cell lysates. Markers of protein molecular weight are indicated on the left side. (**e** and **f**) Relative protein levels of I*κ*B*α* and Gata1 in E14.5 WT and *Stk40* KO fetal liver cell lysates were quantified by the Software Image J. WT: *n*=6; KO: *n*=6. **P≤0.01, ***P≤0.001

As adhesion molecules are known to play critical roles in the EBI formation,^27, 28, 29^ we examined the expression levels of erythroblast macrophage protein (EMP), vascular cell adhesion molecule-1 and intercellular adhesion molecule-1 (ICAM-4). Our quantitative real-time (RT-qPCR) results revealed that deletion of *Stk40* did not affect the mRNA levels of these adhesion molecules (Figure 7b). The result hints that the impaired EBI formation might not stem from compromised expression of the tested adhesion molecules.

### Stk40 regulates TNF-*α* signaling and genes involved in erythropoiesis

To understand how Stk40 participated in erythropoiesis, we compared the transcriptomes of E14.5 fetal livers between WT and *Stk40*^−/−^ embryos by microarray analyses. Among 1295 differentially expressed genes (DEGs), 934 DEGs were upregulated (Figure 7a), implicating a repressive role of Stk40 for gene expression in mouse fetal livers. Consistent with the increased apoptosis of *Stk40*^−/−^ fetal liver cells, microarray analysis revealed 267 genes involved in cell death and proliferation, including *Trp73*, *TGF-β*, *p21* (*Cdkn1a*), *IL21* and *Lcn2.*^7, 30, 31, 32, 33^ Altered expression of these genes was further verified by RT-qPCR (Figure 7b). Interestingly, ingenuity pathway analysis, often used in the identification of upstream regulators of DEGs, of upregulated genes identified TNF-*α* as the most significant upstream regulator (Figure 7c). Indeed, the mRNA level of TNF-*α* increased by about sixfolds in *Stk40* KO liver cells (Figure 7b), implying a potential role of TNF-*α* activation in the *Stk40* deficiency-induced dyserythropoiesis in fetal livers. In total, 133 upregulated DEGs were associated with TNF-*α* signaling, including *Adam8*, *Aldh2*, *Cdkn1a*, *Ccl-6*, *Ccl-20*, *Cxcl-10*, *IFN-γ*, *IL-1b*, *Jun-b* and *Lcn2*. The upregulation of these genes was further verified by RT-qPCR (Figure 7b). TNF-*α* is known to inhibit erythropoiesis and cause anemia in human.^34^ Interestingly, human STK40 was reported as a suppressor of TNF-*α*-induced NF-*κ*B activation.^18^ Therefore, we anticipated that deletion of *Stk40* might lead to over-activation of TNF-*α* signaling in the mouse fetal liver and thus impair erythropoiesis. In support of this, the protein level of I*κ*B*α*, an indicator of the NF-*κ*B activity, was significantly reduced in the *Stk40*^−/−^ fetal livers (Figures 7d and e). Furthermore, in line with a previous report that TNF-*α* could induce erythroid cell death via caspase-mediated cleavage of GATA1,^6^ we observed decreased protein levels of Gata1 and increased cleaved caspase-3 in *Stk40*^−/−^ fetal liver cells (Figures 7d and f). Therefore, our data suggest that deletion of *Stk40* could invoke the aberrant activation of TNF-*α* expression and NF-*κ*B signaling in fetal livers, which may in turn lead to the inhibition of erythropoiesis.

## Discussion

In the present study, utilizing a conventional knockout (KO) mouse model, we report an important role of Stk40 in the fetal liver definitive erythropoiesis, which could be primarily attributed to the disrupted erythroid differentiation and enhanced apoptosis. Our further investigation indicates that aberrantly activated TNF-*α* signaling may underlie impaired erythroid differentiation and survival. Human STK40 was reported to inhibit TNF-*α*-induced NF-*κ*B activation.^18^ In the mouse psoriasis model, miR-31 increases the basal level and TNF-*α*-induced cytokine and chemokine production via targeting Stk40.^35^ This mechanism is also conserved in the endothelial–mesenchymal transition and in associated secretory phenotype induced by TGF-*β*.^36^ Recently, STK40 was reported to suppress the NF-*κ*B pathway involved in human keratinocyte viability, migration and apoptosis.^37^ Therefore, Stk40 may have a conserved inhibitory role of cytokine-induced NF-*κ*B signaling independent of cell types and contexts. Multiple studies have demonstrated that TNF-*α* inhibits erythropoiesis and causes anemia in human.^34, 38, 39, 40, 41^ Moreover, TNF-*α* can induce cell death of erythroid cells via caspase-mediated cleavage of the master regulator of erythropoiesis, GATA1.^9, 15, 42, 43, 44^ Our finding that *Stk40* deletion in the fetal liver causes aberrant activation of TNF-*α* signaling supports the idea that Stk40 plays an important role in repressing TNF-*α* signaling activation. The aberrant activation of TNF-*α* signaling can bring about increased inflammatory cytokine stimulation and subsequent inhibition of erythropoiesis. However, the direct link between Stk40 and TNF-*α* signaling has not been uncovered yet. The transcriptomic and signaling profiles need to be further investigated in purified erythroid subpopulations.

The centrally positioned macrophages in EBIs of the mouse fetal liver also play important roles in promoting erythroblast survival, terminal differentiation and cell cycle progression, as well as enucleation of late-stage erythroblasts.^8, 27, 28, 45^ Several pairs of adhesion molecules are known to play critical roles in EBI formation, including EMP/EMP, *α*4*β*1 integrin/VCAM-1 and ICAM-4/*α*v receptors.^27, 28, 29^ Deletion of *Stk40* did not affect mRNA levels of these adhesion molecules based on our microarray analysis and RT-qPCR analysis. It remains unclear how *Stk40* deletion disrupted the EBI formation.

We found that HSPC compartments in *Stk40*^−/−^ fetal livers were reduced, although the relative frequencies were comparable to those in WT/Het mice due to the significantly reduced liver size. The reduced donor contribution of *Stk40*^−/−^ transplants implies the possibility of a reduced functional HSC pool. The fact that *Stk40*^−/−^ fetal liver cells retained the ability of long-term multi-lineage reconstitution indicates that Stk40 may not play a pivotal role at the HSC level. However, functional HSC populations in recipient mice should be further characterized by signaling lymphocytic activation molecules and other markers.^30^ Transplanted fetal liver cells used in our competition assays contained the mixture of hematopoietic progenitors, matured cells and non-hematopoietic cells, causing the inaccuracy in evaluation of the reconstitution capacity. It remains elusive whether *Stk40*^−/−^ HSCs retain an intact ability of self-renewal. To address this question, FACS-purified fetal liver HSCs and donor-derived HSCs should be used to determine the functionality of Stk40 in serial reconstitutions. Due to the lack of markers to distinguish the origin of erythroid cells, we could not examine the role of Stk40 in the definitive erythropoiesis of donor cells in recipient mice. In the future, ubiquitously expressed reporter genes (e.g., GFP) can be employed to mark donor-derived erythroid cells and thus determine the exact role of Stk40 in BM erythropoiesis. In this study, we focused on the function of Stk40 in definitive erythropoiesis in the mouse fetal livers. However, one can speculate a potential role of Stk40 in the BM and adult erythropoiesis as well. A conditional KO mouse model is currently being developed for dissection of functions of Stk40 in the hematopoietic cell hierarchy and the extrinsic effect of microenvironment.

In summary, our data reveal that Stk40 is required for erythroid differentiation and survival as well as EBI formation in the mouse fetal liver. Identification of cofactors and inhibitors of Stk40 would provide further molecular insights into its functions and develop therapeutic applications for erythropoiesis-related diseases.

## Materials and Methods

### Mice

*Stk40* Het mice were maintained in a C57BL/6J background under specific pathogen-free conditions. All procedures were performed according to the guidelines approved by the Animal Use and Care Committee of Shanghai JiaoTong University School of Medicine. Genotyping was determined by genomic PCR as previously described.^24^

### Hematologic analysis of peripheral blood

For circulating blood cell counts, peripheral blood samples were obtained from the facial vein of E15.5–E18.5 embryos and measured using hematology analyzer Poch-100iv Diff (Sysmex Corp, Kobe, Japan), or yolk sac and umbilical vessels were severed at their attachment to the placenta in PBS containing EDTA. Embryos older than E15.5 were bled with extra decapitation. All blood cells in the dish were collected and counted by Z2 Coulter Counter (Beckman Coulter, CA, USA). Blood smears were prepared and stained with Wright–Giemsa.

### Erythropoietin concentration determination

Whole blood samples were centrifuged at 2000 × *g* for 10 min to pellet blood cells. The plasma was collected and stored at −70 °C until testing. Erythropoietin concentrations were measured by a mouse Erythropoietin Quantikine ELISA Kit (R&D Systems, Minneapolis, MN, USA) as instructed.

### Immunofluorescence staining and flow cytometry analysis

Fetal livers of E14.5 embryos were dissected, minced gently and passed through 40 *μ*m cell strainers (BD Biosciences, San Jose, CA, USA) to obtain single cell suspensions. Cells were incubated with indicated antibodies for 30 min at 4 °C in PBS containing 2% fetal bovine serum. The antibodies used were as follows: Allo-phycocyanin (APC)-anti-Ter119 (TER-119), Phycoerythrin APC-anti-B220 (RA3-6B2), PE-anti-CD3e (145-2C11), Biotin-anti-CD3e (145-2C11), Biotin-anti-CD4 (GK1.5), Biotin-anti-CD8 (SK1), Biotin-anti-Gr-1 (RB6-8C5), Biotin-anti-Mac-1 (M1/71), Biotin-anti-Ter119 (TER-119), Biotin-anti-B220 (RA3-6B2), APC-Cy7 Streptavidin, APC-anti-c-Kit (2B8), PE-Cy7-anti-Sca1 (D7), FITC-anti-CD34 (RAM34), PE-anti-CD16/32 (2.4G2), PE-anti-IL7R*α* (SB/199) and FITC-Annexin V (BD Biosciences). The flow cytometry analyses were performed with BD FACS Aria II or Accuri C6 flow cytometers.

### CFU analyses

The CFU-E and BFU-E of WT and *Stk40*^−/−^ E14.5 fetal liver cells were analyzed according to instructions of manufacture (Stemcell Technologies, Vancouver, BC, Canada). Erythroid, myeloid and mixed lineage colonies were counted based on morphological criteria. The MethoCult M3334 medium was used for CFU-E, and M3434 for BFU-E, CFU-G, CFU-M, CFU-GM and CFU-GEMM.

### Competitive reconstitution assays

The Ly5 congenic mouse system was used to perform competitive reconstitution assays. In the primary transplantation, 5 × 10^5^ fetal liver nucleated cells (Ly5.2/CD45.2) were mixed with the same number of BM competitor cells (Ly5.1/CD45.1) and injected intravenously into recipient mice lethally irradiated at a dose of 9.5 Gy. Cells from the peripheral blood and BM were stained with fluorescence-conjugated antibodies for population analysis at indicated time points. Eighteen weeks after transplantation, 1 × 10^6^ BM nucleated cells from killed primary recipients were transplanted into the secondary recipient mice. Cells from the secondary recipients were timely analyzed as in the primary transplantation.

### RNA extraction and RT-qPCR

Total RNA was extracted using the TRIzol reagent (Invitrogen/Life Technologies, Grand Island, NY, USA) in accordance with the manufacturer's instructions. Reverse transcription was performed with a Fastquant reverse kit (Tiangen, Beijing, China). RT-qPCR was carried out on ABI 7900 using the Roche FastStart Universal SYBR Green Master (Rox). Primers used in this study are provided in Supplementary Table 1.

### RNA microarray analyses

Total fetal liver RNA was isolated as described above. Each sample contained pooled RNA from six livers of E14.5 embryos of the same genotype. Two biological replicates for each genotype were prepared and hybridized to the Affymetrix mouse 430 2.0 array by the Shanghai Biochip Company. For data processing, first, all probe-sets with negative values in all samples were removed; second, probe-set values inconsistent in each group (WT or KO) were also removed; third, values less than 0.1 were set to 0.1 for further analysis. DEGs were defined by a fold change of two. DEGs occurred in both decreased and increased list were removed as well. The heat map was drawn by R package ‘heatmap.2'. Upstream pathways were analyzed by ingenuity pathway analysis (QIAGEN). Microarray raw data were submitted to GEO (GSE95017) and processed data are provided in Supplementary Table 2.

### Protein preparation and western blotting

Whole cell lysate was collected in the lysis buffer (2 mM EDTA, 0.5% NP-40, 50 mM Tris-HCl, pH 7.5, 150 mM NaCl with protease inhibitors) and quantified using a BCA kit (Thermo Scientific, Wilmington, DE, USA). Relative protein levels were quantified by the software Image J (NIH, Bethesda, MD, USA).

### EBIs

Native EBIs were isolated from E13.5 or E14.5 fetal livers as reported.^45^ For island reconstitution, erythroblasts were stripped from adherent macrophages with Dulbecco's PBS (Ca^2+^/Mg^2+^ free), and erythroid cells from other embryos with the indicated genotype were added to the stripped macrophages. Native or reconstituted clusters were dipped in the RPMI 1640 medium (Thermofisher Scientific, MA, USA) to remove non-adherent cells. Islands were fixed in 4% PFA in PBS for 20 min, stained with APC-anti-Ter119, FITC-anti-F4/80 and Hoechst 33342 and imaged by a confocal microscopy (ZEISS 710) with a × 63 oil immersion objective (N.A. 1.4).

### Statistical analysis

Results are shown as mean±S.D. The significance of differences was assessed with a two-tailed unpaired Student *t*-test. *P≤0.05, **P≤ 0.01 and *** *P*≤0.001. Prism version 6.0c software (Graph Pad Software) was used for statistical evaluation.
