# Supplementary material for: Deletion of Stk40 impairs definitive erythropoiesis in the mouse fetal liver
Source: Cell Death Dis. 2017 Mar 30;8(3):e2722–. doi: 10.1038/cddis.2017.148 (PMC5386544; doi:10.1038/cddis.2017.148)
Supplement: Supplementary Information [file cddis2017148x1.docx]

**Supplementary Figure legends**

**Supplementary Figure 1. Definitive erythropoiesis is impaired in *Stk40^-/-^* fetal livers.**

(a) Numbers of white blood cells (WBCs) in peripheral blood of *Stk40* WT, Het and KO embryos at E18.5.

(b) Numbers of platelets (PLTs) in peripheral blood of *Stk40* WT, Het and KO embryos at E18.5. For panels (a) and (b), WT, n=26; Het, n=48; KO, n=17. ns, no significance.

(c) Cellularity of circulating blood cells from E13.5 to birth of *Stk40* WT/Het and KO mice. E13.5: WT, n=6; Het, n=11; KO, n=7. E14.5: WT, n=6; Het, n=9; KO, n=6. E15.5: WT, n=6; Het, n=20; KO, n=5. E18.5: WT, n=26; Het, n=42; KO, n=16. * P ≤ 0.05, *** P ≤ 0.001.

(d) Cellularity of circulation blood cells per body weight (gram) from E16.5 to E18.5 WT, Het and *Stk40* KO embryos. E16.5: WT, n=14; Het, n=14; KO, n=7. E18.5: WT, n=5; Het, n=12, KO, n=6. ** P ≤ 0.01, *** P ≤ 0.001.

(e) Representative peripheral blood smears of E18.5 WT and *Stk40* KO embryos stained with Wright-Giemsa.

(f) Gross morphology of WT and *Stk40* KO embryos at E13.5. White arrowheads indicate subcutaneous edema. Black arrows indicate the position of the fetal liver. Scale bar, 3 mm.

(g) Relative cellularity of fetal livers per body weight (gram) from E12.5 to E16.5 of *Stk40* WT/Het and KO embryos. E12.5: WT/Het, n=18; KO, n=7. E13.5: WT/Het, n=44; KO, n=21. E14.5: WT/Het, n=43; KO, n=17. E16.5: WT/Het, n=41; KO, n=14. ** P ≤ 0.01, *** P ≤ 0.001; ns, no significance.

(h) Representative flow cytometry plots of monocytes (Gr-1^-^Mac-1^+^) and granulocytes (Gr-1^+^Mac-1^+^) from *Stk40* WT/Het and KO embryos at E14.5.

(i) Frequencies of granulocytes (Gr-1^+^Mac-1^+^) and monocytes (Gr-1^-^Mac-1^+^) from *Stk40* WT/Het and KO embryos at E14.5 shown in (h).

(j) Frequencies of B cells (B220^+^) and T cells (CD3^+^) from *Stk40* WT/Het and KO embryos at E14.5.

(k) Absolute numbers of B cells (B220^+^) and T cells (CD3^+^) from *Stk40* WT/Het and KO embryos at E14.5. For panels (h) – (k), WT/Het, n=38; KO, n=14. Ns, no significance.

**Supplementary Figure 2. The representative images of BFU-E (a), CFU-E (b), CFU-GEMM (c), CFU-G (d), CFU-M (e) and CFU-GM (f) from E14.5 wild type or *Stk40* KO fetal liver cells.**

The same initial fetal liver cells from E14.5 wild type and *Stk40* KO embryos were seeded into M3334 and M3434 media, respectively, then incubated in 37^o^C with 5% CO_2_ atmosphere. The numbers of CFU-E, BFU-E, CFU-G, CFU-M, CFU-GM and CFU-GEMM colonies were determined based on morphological criteria.

**Supplementary Figure 3. Flow cytometry analysis of hematopoietic stem and progenitor cells of E14.5 wild type and *Stk40* KO fetal livers.**

Fetal liver cells were stained with antibodies as indicated to mark LSK, MEP, CMP and GMP. Representative images of flow cytometry plots are displayed.

**Supplementary Figure 4. Analysis of hematopoiesis of the secondary transplantation**

(a) Frequencies of donor (E14.5 WT and *Stk40* KO fetal livers)-derived cells in recipient BM after 18 weeks of the primary transplantation.

(b)-(e) Frequencies of donor derived B lymphoid (B220, b), T lymphoid (CD3, c), granulocyte (Gr-1^+^Mac-1^+^, d) and monocyte (Gr-1^-^Mac-1^+^, e) in peripheral blood (PB) cells after the secondary transplantation.

(f) Mean ratios of donor chimerism of WT and *Stk40* KO cells at different time points of the primary and secondary transplantation.

(g) Frequencies of donor cells in the BM at 18 weeks after the secondary transplantation.

(h) Frequencies of donor derived CMPs, GMPs and MEPs in the BM at 18 weeks after the secondary transplantation.

For all panels, n=12 for both WT and *Stk40* KO embryos. * P ≤ 0.05, ** P ≤ 0.01, *** P ≤ 0.001; ns, no significance.

**Supplementary Figure 5. Apoptosis analysis for MEPs in the E14.5 fetal liver cells.**

(a) Pepresentative plots of flow cytometry analysis of apoptosis in MEPs by staining with Annexin V.

(b) Frequencies of apoptotic fetal liver MEPs (Annexin V^+^) from E14.5 *Stk40* WT/Het and KO embryos. WT/Het, n=19; KO, n=19. ns, no significance.

**Supplementary Figure 6. The representative TEM images of fetal livers from E14.5 WT and *Stk40* KO embryos.**

The erythroblastic island (EBI) is mainly composed of one centrally positioned macrophage and several surrounding erythroblasts at different differentiation stages. Its morphology resembles a ‘rosette’ under TEM. The typical rosette organization of EBIs in WT fetal livers and disorganized EBIs in *Stk40* KO livers at E14.5 is shown. M, macrophages; E, erythroblasts; G, granulocytes. Scale bar, 5 μm.

Supplementary Table 1. List of real time PCR (qPCR) primers used in this study.

Supplementary Table 2. Processed microarray data of both wild type and *Stk40* knockout E14.5 fetal liver cells.
