# Supplementary figures and images for: Deletion of Stk40 impairs definitive erythropoiesis in the mouse fetal liver
Source: Cell Death Dis. 2017 Mar 30;8(3):e2722–. doi: 10.1038/cddis.2017.148 (PMC5386544; doi:10.1038/cddis.2017.148)

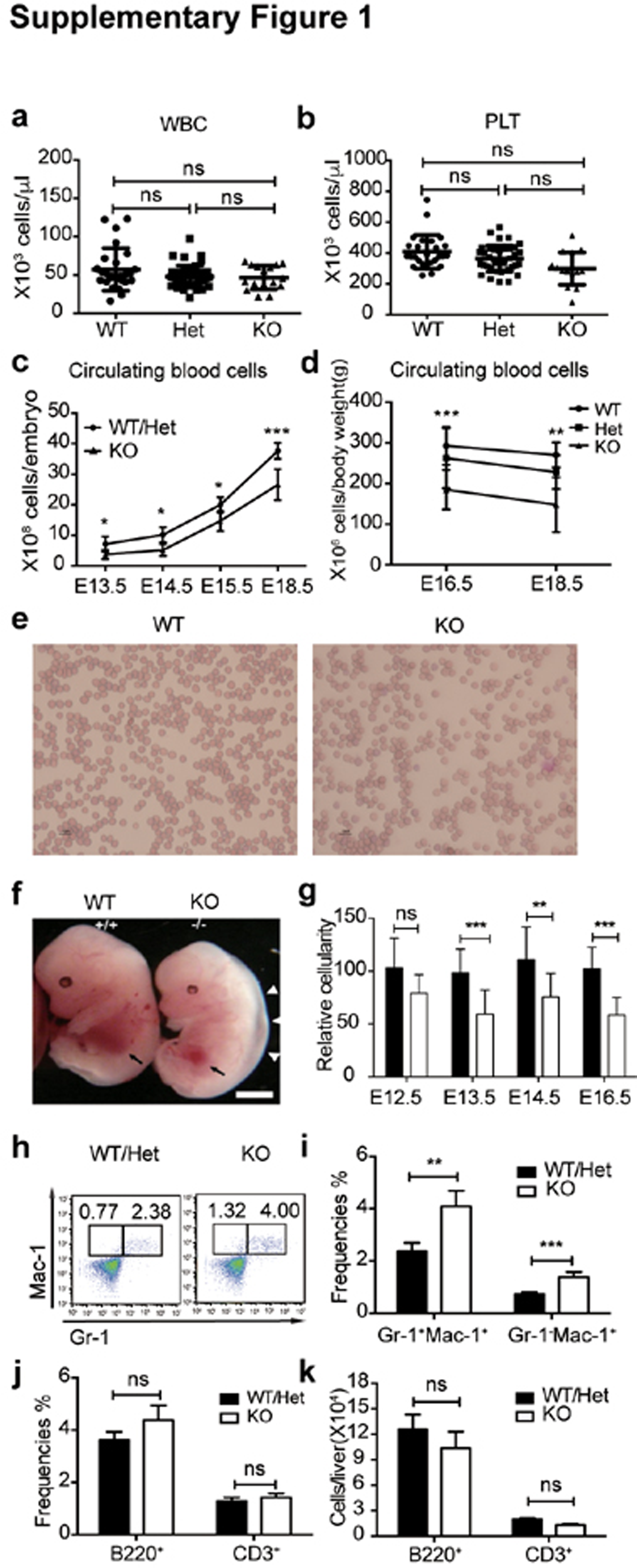

Supplement: Supplementary Figure 1 [file cddis2017148x2.tif]

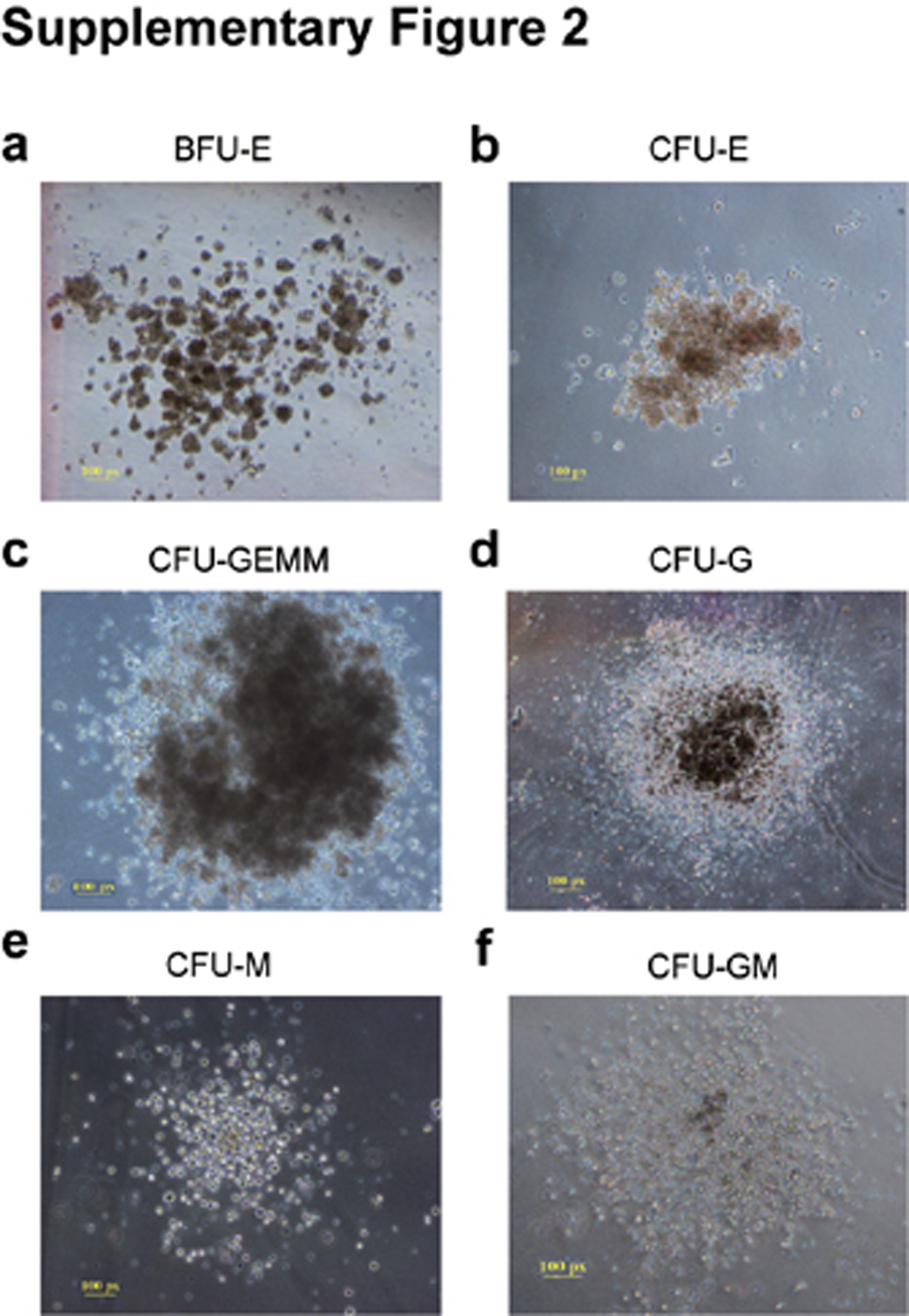

Supplement: Supplementary Figure 2 [file cddis2017148x3.tif]

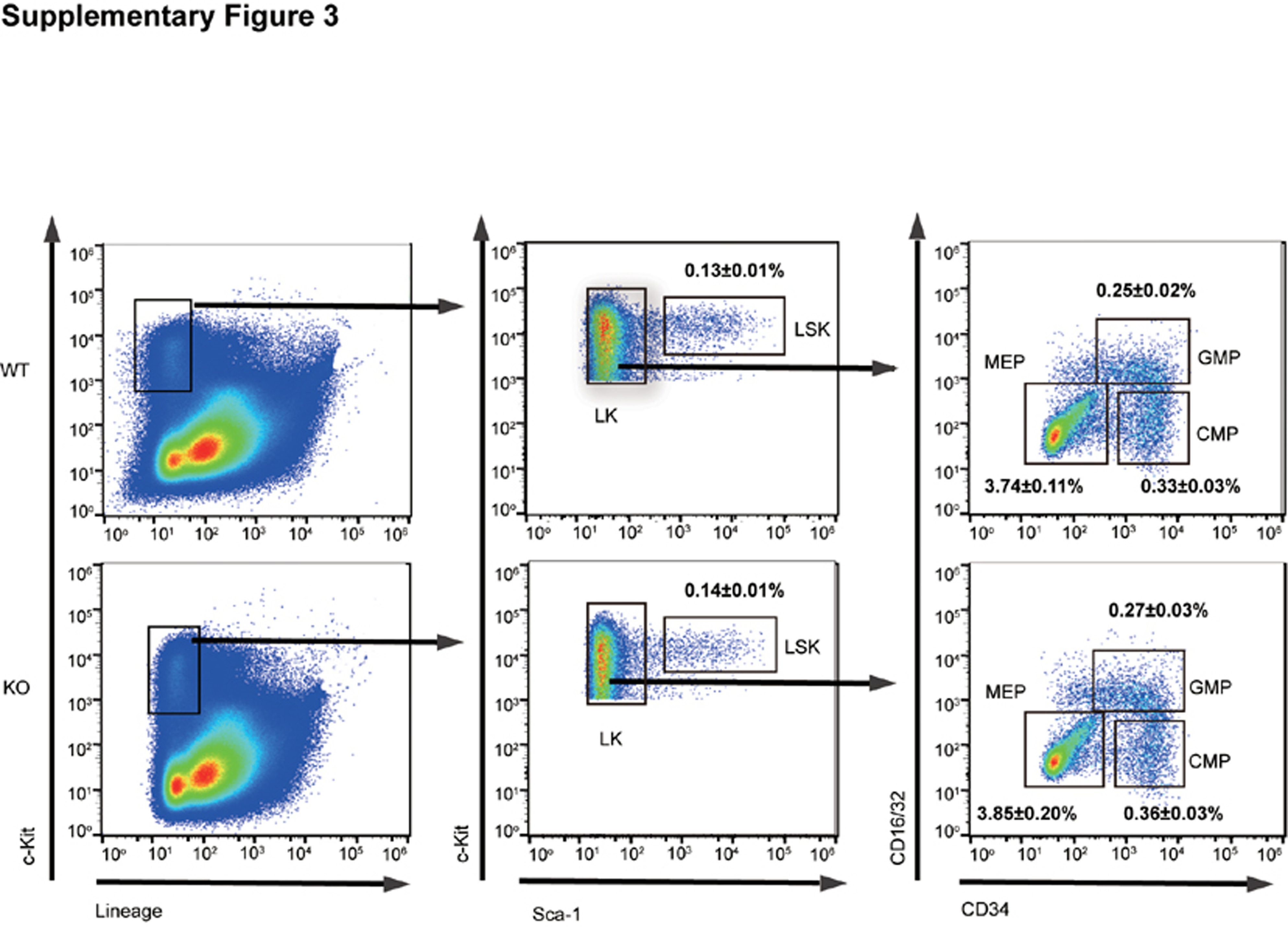

Supplement: Supplementary Figure 3 [file cddis2017148x4.tif]

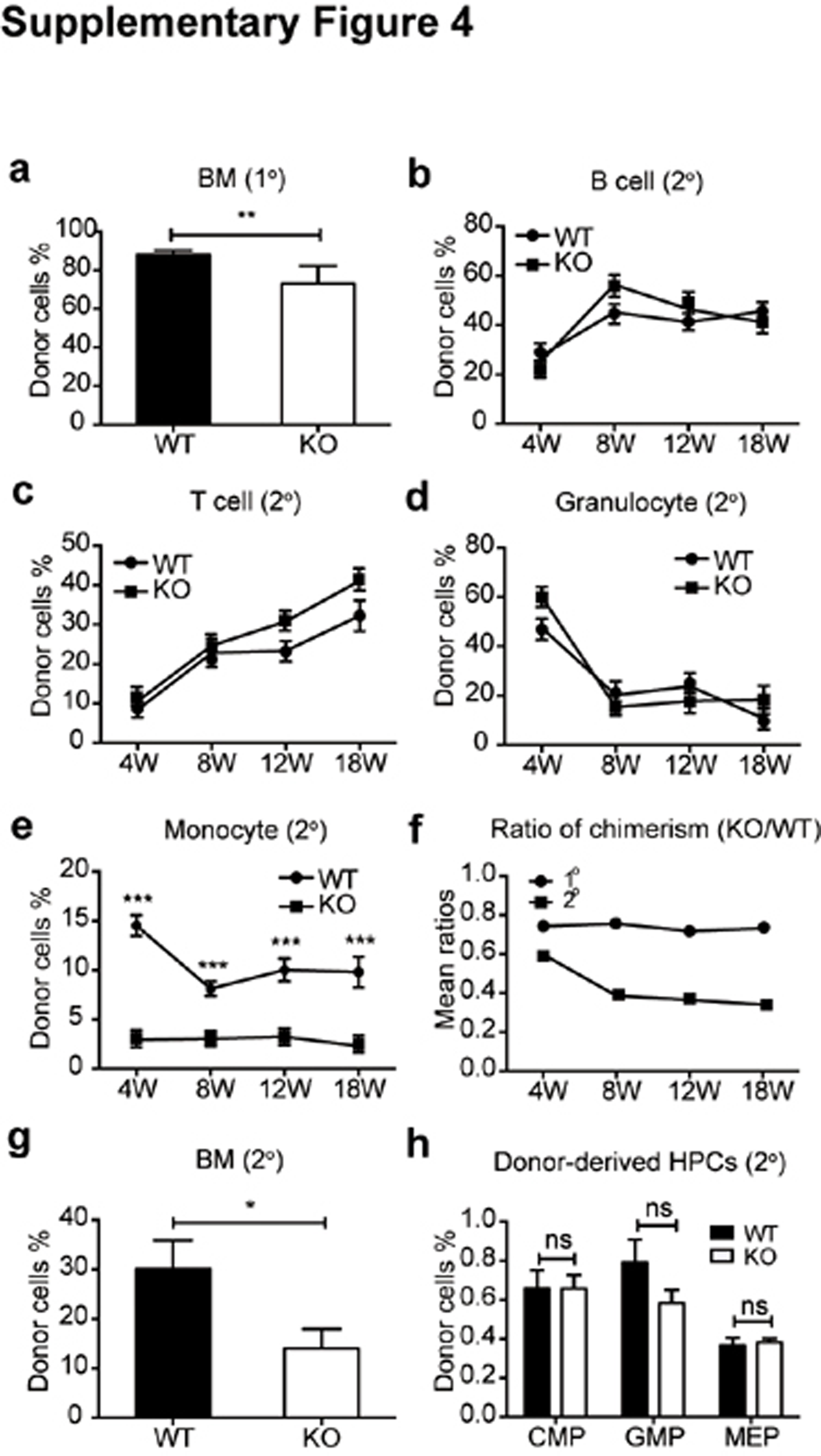

Supplement: Supplementary Figure 4 [file cddis2017148x5.tif]

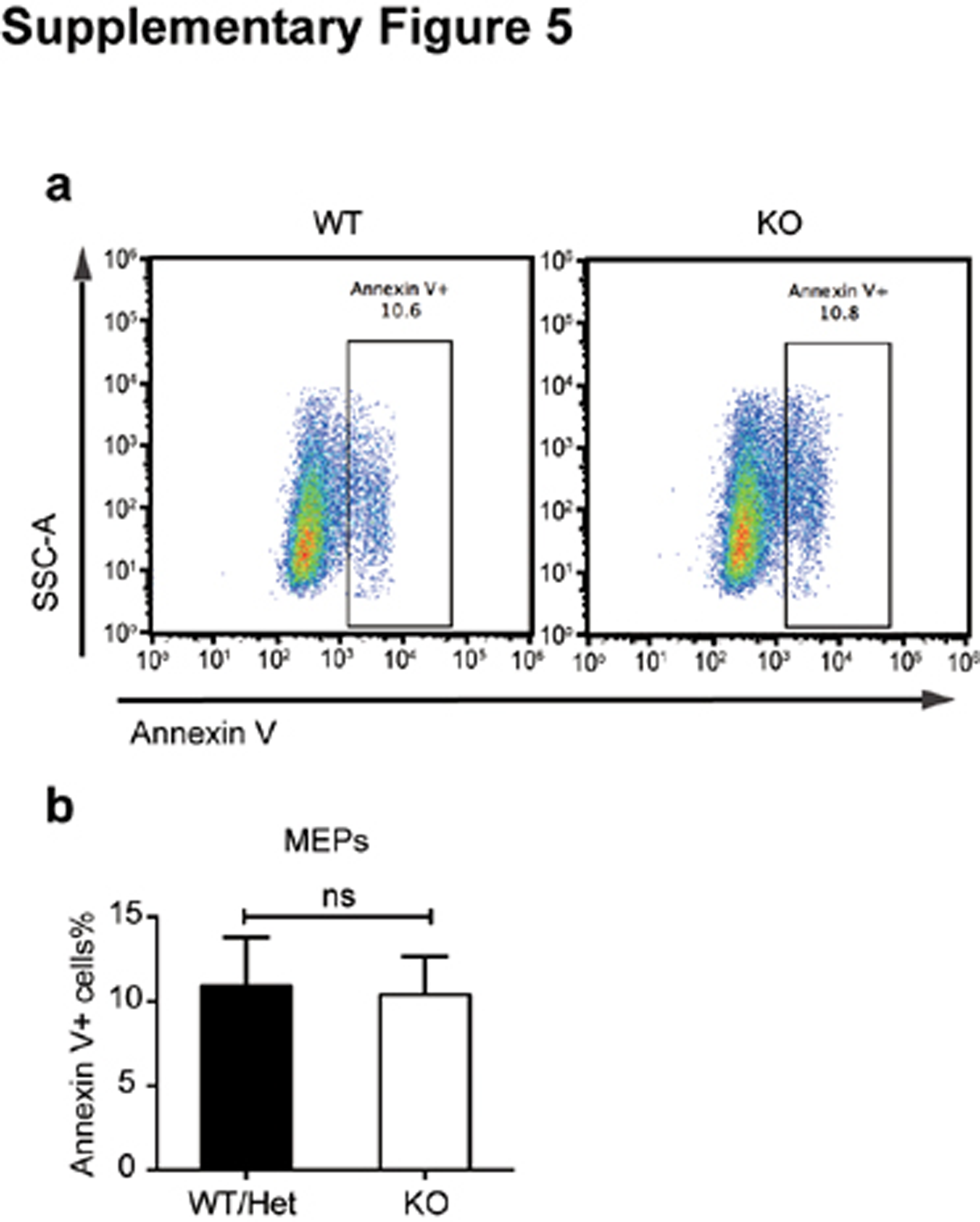

Supplement: Supplementary Figure 5 [file cddis2017148x6.tif]

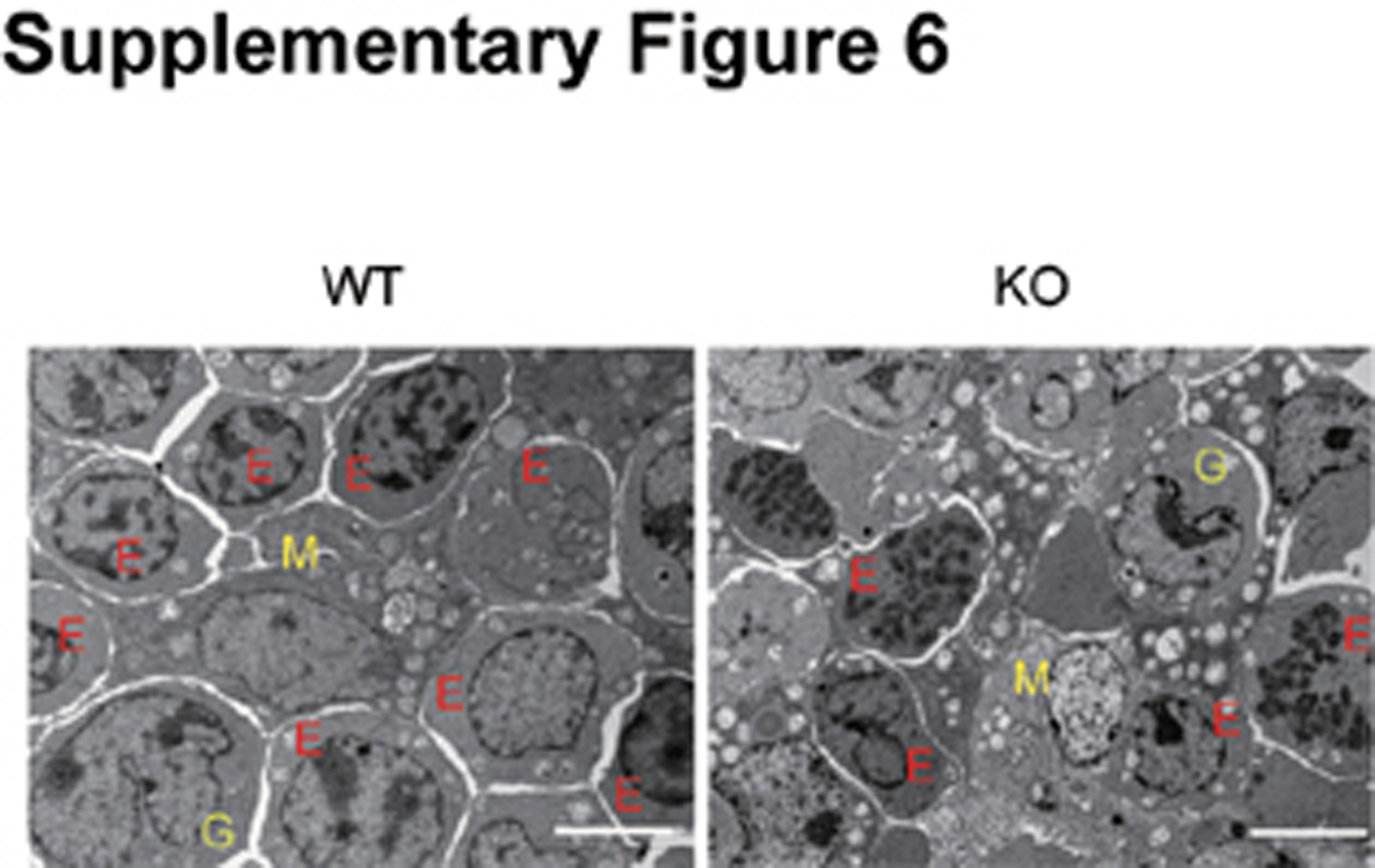

Supplement: Supplementary Figure 6 [file cddis2017148x7.tif]
