# Supplementary material for: Deletion of Stk40 impairs definitive erythropoiesis in the mouse fetal liver
Source: Cell Death Dis. 2017 Mar 30;8(3):e2722–. doi: 10.1038/cddis.2017.148 (PMC5386544; doi:10.1038/cddis.2017.148)
Supplement: Supplementary Table 1 [file cddis2017148x8.docx]

**Supplementary Table 1**

The mRNA from E14.5 fetal liver nucleated cells of WT and *Stk40* KO embryos was extracted and reversed as described above. Real time PCR (RT-qPCR) was performed using the primers listed below:

| Gene | Forward primer (5' to 3') | Reverse primer (5' to 3') |
| --- | --- | --- |
| Adam8 | AGTTCCTGTTTATGCCCCAAAG | AGTTCCTGTTTATGCCCCAAAG |
| Aldh2 | GACGCCGTCAGCAGGAAAA | CGCCAATCGGTACAACAGC |
| Cdkn1a | CCTGGTGATGTCCGACCTG | CCATGAGCGCATCGCAATC |
| Ccl-6 | GCTGGCCTCATACAAGAAATGG | GCTTAGGCACCTCTGAACTCTC |
| Ccl-20 | GCCTCTCGTACATACAGACGC | CCAGTTCTGCTTTGGATCAGC |
| Cxcl10 | CCAAGTGCTGCCGTCATTTTC | GGCTCGCAGGGATGATTTCAA |
| Gapdh | AGGTCGGTGTGAACGGATTTG | TGTAGACCATGTAGTTGAGGTCA |
| IFN-γ | ATGAACGCTACACACTGCATC | CCATCCTTTTGCCAGTTCCTC |
| IL-1b | GCAACTGTTCCTGAACTCAACT | ATCTTTTGGGGTCCGTCAACT |
| IL-21 | GGACCCTTGTCTGTCTGGTAG | TGTGGAGCTGATAGAAGTTCAGG |
| Jun-b | TCACGACGACTCTTACGCAG | CCTTGAGACCCCGATAGGGA |
| Lcn2 | TGGCCCTGAGTGTCATGTG | CTCTTGTAGCTCATAGATGGTGC |
| Stk40 | GGCATTGCTGGAAATAACGCA | CCCCTCTCCTCAAGGGTGA |
| TGF-β | TCGACATGGATCAGTTTATGCG | CCCTGGTACTGTTGTAGATGGA |
| Trp73 | GCACCTACTTTGACCTCCCC | GCACTGCTGAGCAAATTGAAC |
| TNF-α | CCTGTAGCCCACGTCGTAG | GGGAGTAGACAAGGTACAACCC |
| Kit | CTCCCCCAACAGTGTATTCAC | TAGCCCGAAATCGCAAATCTT |
| ICAM-4 | GGGCCACAAGTACACTCTGC | ACGTAGGTCAAAGTGACATTAGC |
| VCAM-1 | AGTTGGGGATTCGGTTGTTCT | CCCCTCATTCCTTACCACCC |
